# Local and collective transitions in sparsely-interacting ecological communities

**DOI:** 10.1371/journal.pcbi.1010274

**Published:** 2022-07-11

**Authors:** Stav Marcus, Ari M. Turner, Guy Bunin

**Affiliations:** Department of Physics, Technion-Israel Institute of Technology, Haifa, Israel; Abdus Salam International Centre for Theoretical Physics, ITALY

## Abstract

Interactions in natural communities can be highly heterogeneous, with any given species interacting appreciably with only some of the others, a situation commonly represented by sparse interaction networks. We study the consequences of sparse competitive interactions, in a theoretical model of a community assembled from a species pool. We find that communities can be in a number of different regimes, depending on the interaction strength. When interactions are strong, the network of coexisting species breaks up into small subgraphs, while for weaker interactions these graphs are larger and more complex, eventually encompassing all species. This process is driven by the emergence of new allowed subgraphs as interaction strength decreases, leading to sharp changes in diversity and other community properties, and at weaker interactions to two distinct collective transitions: a percolation transition, and a transition between having a unique equilibrium and having multiple alternative equilibria. Understanding community structure is thus made up of two parts: first, finding which subgraphs are allowed at a given interaction strength, and secondly, a discrete problem of matching these structures over the entire community. In a shift from the focus of many previous theories, these different regimes can be traversed by modifying the interaction strength alone, without need for heterogeneity in either interaction strengths or the number of competitors per species.

## Author summary

We study how sparse interactions—where each species interacts significantly with only a few others—shape species-rich ecological communities. In a theoretical model where a community is assembled from a species pool, we find that the community characteristics strongly depend on the interaction strength, even when it has no heterogeneity. For strongly competitive interactions, the network of interactions of the coexisting species breaks up into many disconnected components. These grow larger for weaker interactions, until finally reaching a single component encompassing all species. Changes in the allowed shapes of these network components are accompanied by sharps jumps in diversity and other properties. In addition to these jumps, we find a percolation transition in the network of coexisting species, where most species coalesce into a single giant component, and a transition between a unique and many alternative states. Many of these characteristics are unique to sparsely interacting networks, and may allow to identify and make predictions on such communities through their macro-ecological properties.

## 1 Introduction

Interactions between species play important roles in shaping ecological communities. A central challenge in community ecology is to relate properties of interactions, such as their strength and organization, to characteristics of communities such as diversity and response to perturbations. In modeling, theory, and simulations, some of the potential interactions are assumed to be negligible or irrelevant and are taken to be zero, a property known as sparseness.

Broadly speaking, theoretical approaches vary with the fraction of all potential interactions that are kept in the network. When each species interacts significantly with only a few others, studying the structure of the sparse network of remaining interactions has been fruitful [1]. Many phenomena have been studied, including extended properties such as percolation, and more local properties, such as the distribution of degree (number of species interacting with each species). An extensive body of work looks at local patterns within the network [1–5]. Central and on-going questions within this line of investigation include: whether these local patterns are more common than some null expectation; whether they play a functional role [6, 7]; whether a bottom-up approach connecting local properties to ecosystem-level properties such as diversity is possible [8, 9]; and whether the ignored “weak” links can indeed be neglected [10].

In the other limit, when many or all possible interactions are present, techniques have been developed [8, 11–19] that relate the interaction strengths to properties such as the diversity, existence of multiple stable states, and persistent dynamics. Here two approaches have been used to model the community. In one, the dynamics is linearized around a fixed point, and the parameters describing the dynamics of coexisting species are sampled at random. This approach predicts stability bounds [11, 14], and has also been applied to sparse interactions [20].

In the other approach, known as community assembly, the dynamics of species from a regional species pool is run, possibly resulting in the extinctions of some of the species. One interesting observation within the assembly approach, is that there are sharp transitions in many-species communities, where persistent fluctuations, very many alternative equilibria, or other properties emerge abruptly as relevant interaction characteristics are changed [8, 11–13, 15, 17, 18]. These characteristics are, for example, moments of interaction strengths distribution [21]. Such transitions are known as collective transitions, because they arise from community-wide processes, and a result of this is that they become sharp in the many-species limit. Whether and how these phenomena are found when interactions are very sparse (with a finite number of links per species), and whether they are at all related to local connectivity patterns that have been discussed for sparse systems, has received little attention.

**Fig 1:**
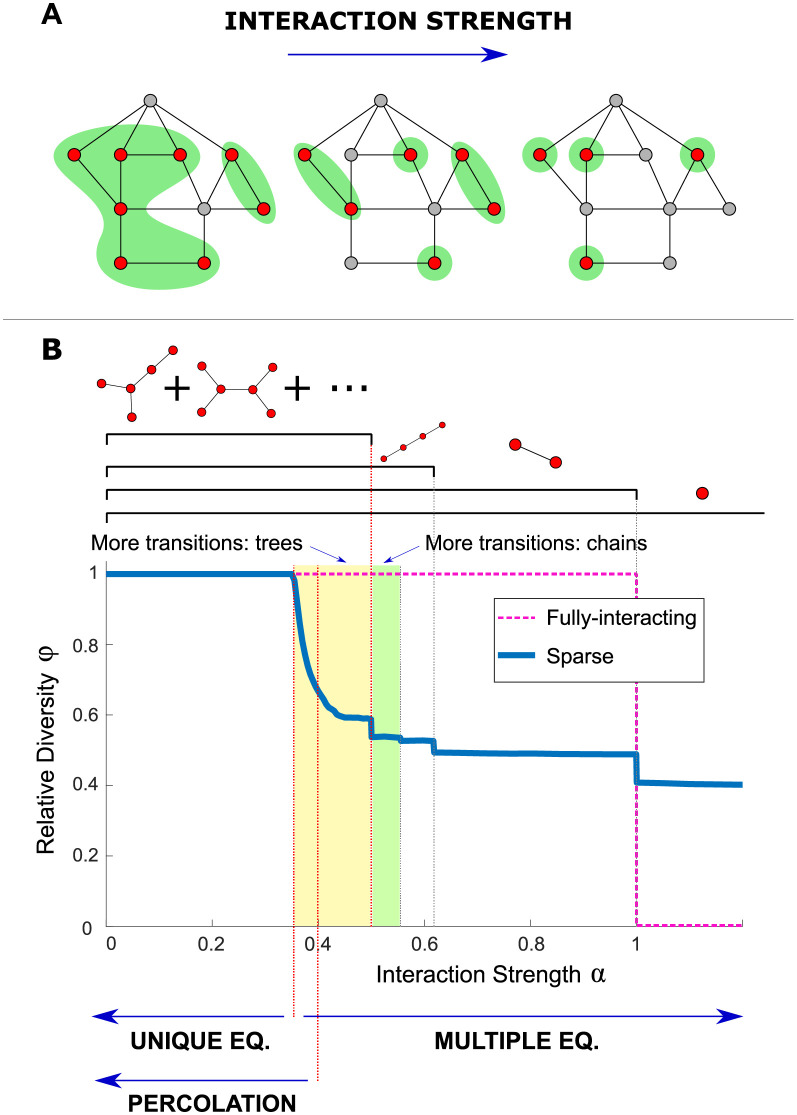
Transitions in community structure. (A) Equilibria reached at different interaction strengths. Red vertices represent persistent species, gray extinct species, and edges mark pairs of interacting species. The persistent species can be divided into connected subgraphs, shown with green background, separated by the extinct species. As the interaction strength *α* is increased, there are fewer and typically smaller allowed subgraphs, reducing the number of coexisting species. (B) The relative diversity *ϕ* = S*/*S* (where S* is the number of persistent species) at equilibrium, from simulations with pool size *S* = 400 and sparse interactions with degree *C* = 3 (blue); and for comparison for a fully-interacting community (pink, dashed), which exhibits only a single jump at *α* = 1. The sparse case exhibits infinitely-many sharp transitions, some of which are marked by dashed vertical lines. By order of increasing *α*, the first transition is at $\alpha_{\text{UE}}=\frac{1}{2\sqrt{2}}$, from a unique to multiple equilibria. Next there is a percolation transition at *α*_perc_ ≈ 0.41, below which a finite fraction of the persistent species belong to a single giant connected component. Above this value are seen several jumps. These result from changes in the allowed connected subgraphs, specifically those that are trees. At $\alpha_{\text{chain}}^{\left(\infty \right)}=\frac{1}{2}$ trees more complicated than a linear chain stop being allowed; there are three more visible jumps corresponding to the ruling out of the length 6, 4, and 2 chains, with other similar jumps that are too small to be seen by eye (green region). There are also many jumps associated with trees that are not chains (yellow region).

Here we find that sparsely-interacting communities can exhibit phenomena that have been the focus of both lines of investigation, in different regimes, depending on interaction strength. We study a theoretical model where a community is assembled from a species pool. To understand the structure of its equilibria, we consider a graph whose vertices include only the species present in the community. We find that when interactions are strong, this graph is composed of many disconnected components. These connected subgraphs, illustrated in Fig 1A, are central to our theory. In the strongly-competitive limit, no two interacting species can coexist, and so the connected components of the graph will be single species [8, 22]. The number of possible connected subgraph structures grows as the interaction strength is lowered, with the subgraphs typically increasing in size. The topology of these finite size subgraphs plays a defining role in coexistence: the problem of species coexistence reduces to a discrete problem on graphs involving local rules, in the spirit of previous works focused on the role of local network patterns. The addition of each new allowed structure is marked by a transition in diversity and species abundance distributions. Note that this would not be possible in a fully-interacting community, which cannot break into multiple connected subgraphs.

At lower interaction strengths, as the interaction strength is varied, we find a percolation transition and a transition between unique and multiple alternative equilibria, similar to ones found in fully-interacting systems [23, 24].

Interestingly, none of these phenomena require heterogeneity in either the degree or the strength of interactions. In fact, the interactions may even be locally ordered, that is, almost all species can have identical neighborhoods up to a finite distance in the network. This is in contrast to collective transitions studied previously, in which heterogeneity is necessary for the transitions to occur [8, 11–17]. The interaction strength thus becomes an important parameter on its own, divorced from the width of the distribution.

The paper is organized as follows. Sec 2.1 introduces a theoretical model of a sparsely interacting competitive community assembled from a species pool, in which each species interacts with the same number of other species, and all interaction are of identical strength. The properties of equilibria at different interaction strengths are discussed. Interacting subgraphs of coexisting species are introduced and their role is elucidated. Jumps in diversity, a percolation transition and a unique- to multiple-equilibria transition are found. Sec 2.2 extends the model to include heterogeneity in the network of the vertex degree and interaction strengths. Sec 2.3 shows how connected subgraphs form by combinations of smaller ones. Sec 3 concludes with a discussion.

## 2 Methods and results

### 2.1 Constant sparse interactions

#### 2.1.1 The model

We work within the framework of species assembly, where species migrate from a species pool, and interact inside a community. The abundances change in time according to the standard multi-species Lotka-Volterra equations. There are *S* species in the pool. The abundance of the *i*-th species, *N*_*i*_, follows the equation

$$\begin{array}{c} \frac{dN_{i}}{dt}=r_{i}N_{i}(1-\sum_{j}\alpha_{ij}N_{j})+\lambda_{i}. \end{array}$$ (1)

where *α*_*ij*_ are the interaction coefficients, *r*_*i*_ the growth rates, and λ_*i*_ the migration rates.

In this paper the matrix *α*_*ij*_, called the interaction matrix [25, 26], is always assumed to be symmetric, *α*_*ij*_ = *α*_*ji*_, with equal intraspecific competition for all species, *α*_*ii*_ = 1. The symmetry ensures that the dynamics in Eq (1) always reaches an equilibrium [27]; There may be one or more such equilibria. Here we only consider competitive interactions, *α*_*ij*_ ≥ 0, and assume that all growth rates are positive, *r*_*i*_ > 0; other than that the values of the *r*_*i*_’s have no effect on the set of stable equilibria. In simulations we take all *r*_*i*_ = 1, and run Eq (1) until changes in the *N*_*i*_-values are small. The migration strengths λ_*i*_ are taken to be small, λ_*i*_ → 0^+^, ensuring that at an equilibrium (i.e. a stable fixed point), all species that could invade do so. We use a migration rate of λ_*i*_ = 10^−10^, and species are considered extinct when *N*_*i*_ < 10^−5^. To ensure a true equilibrium has been reached, we separate the species considered extinct from those considered present. We then verify that the present species satisfy 1 − ∑_*j*_ *α*_*ij*_ *N*_*j*_ = 0 among themselves for all *i*, and each extinct species *i* cannot invade, *dN*_*i*_/*dt* < 0. Further details of the simulation procedure appear in Section F in S1 Text.

We are interested here in sparse interactions, where many of the pairs of species do not interact (*α*_*ij*_ = 0). The network of interactions forms an undirected graph, with vertices representing species and edges representing pairs of interacting species, sometimes called the community graph [28].

It is common to use random interactions sampled from different distributions, which capture different interaction characteristics. In this section we will consider the following model: (1) Each species interacts with exactly *C* other species, with the interacting pairs chosen at random so that the community graph is a random *C*-regular undirected graph. (2) The interaction strength is equal for all interacting pairs. Therefore, the interaction matrix can be written as *α*_*ij*_ = *δ*_*ij*_ + *αA*_*ij*_, where *α* is the interaction strength, and *A*_*ij*_ is the symmetric adjacency matrix of the community graph. We consider *C* ≪ *S*, and more precisely the limit of large *S* at constant *C*. We will see that this simplified model already yields dramatically different results as compared with a fully-connected system with all-equal interactions. Extensions to varying interaction strength and number of interaction per species are then discussed in Section 2.2.

From a physics perspective, this model is related to models of antiferromagnetic interactions on a tree [29–32]. However, many of our results arise from the distinct features of interacting populations, with species abundances described by continuous, non-negative variables, with zero being special (extinct species).

We limit the discussion to properties of the system’s equilibria, and not the dynamics towards the equilibria, or under additional noise, which are very interesting (some already discussed in [33]) but beyond the scope of this work.

#### 2.1.2 Overview of different regimes

To get a bird’s eye view of the different behaviors, we follow the diversity at the equilibria as a function of the interaction strength *α* (recall that in this first model *α* is identical for all pairs). Denote by *S* the total number of species in the pool, S* the number of coexisting species at an equilibrium (species richness), and define the relative diversity *ϕ* = S*/*S*. Fig 1 shows simulation results for *ϕ* as a function of *α*. *ϕ* is estimated by running simulations of Eq (1) over many realizations of adjacency matrices *A*_*ij*_, starting from a few different initial conditions per realization, with each initial abundance *N*_*i*_(*t* = 0) sampled uniformly from [0, 1]. The variability in *ϕ* between simulations under the same conditions decreases with the diversity *S*, and for large *S* it is essentially set deterministically. In this limit *ϕ* also becomes independent of *S*, as illustrated in Fig A in S1 Text.

For comparison, the case of a full interaction matrix, where all species interact with each other with strength *α* is also plotted. In this case the behavior is simple: For *α* < 1 there is a unique fixed point in which all species are persistent with equal abundances, so *ϕ* = 1, while for *α* > 1 there are *S* different fixed points, each with a single persistent species so that *ϕ* = 1/*S*, tending to zero at large *S*. For both the fully-interacting system and a sparse system on a random regular graph, the interactions are locally ordered (the neighborhood of any vertex is a tree with probability 1, see next section), and both admit a fixed point with all species present. The sparsely-interacting system, in contrast, is very rich and exhibits multiple different behaviors with sharp transitions between them. At values of *α* close to zero, the interactions are weak enough to allow all species to coexist with *ϕ* = 1, again with all equal abundances. This persists for larger *α* up to some critical value *α*_UE_ where *ϕ* starts to decrease, above which this fixed point is no longer stable. At a higher value *α*_perc_ there is a percolation transition, above which none of the components of persistent species scales with the system size *S*. The relative diversity *ϕ* keeps decreasing until it reaches another transition where there is a jump in *ϕ*, at a value we denote by $\alpha_{\text{chain}}^{\left(\infty \right)}$. At $\alpha >\alpha_{\text{chain}}^{\left(\infty \right)}$, the relative diversity *ϕ*(*α*) consists of infinitely many plateaus punctuated by jumps, until the last jump at *α* = 1 and a single plateau above it.

In the following sections, we discuss this behavior in detail, and explain the multiple changes in system behavior and the reasons behind them. We will show in the next sections that $\alpha_{\text{UE}}=\frac{1}{2\sqrt{C-1}}$ and $\alpha_{\text{chain}}^{\left(\infty \right)}=\frac{1}{2}$, and provide analytical values for *α* of all jumps in *ϕ*(*α*) at *α* ≥ 1/2. In Subsection 2.1.4 we discuss the percolation transition, and in Subsection 2.1.5 the unique to multiple equilibria transition, and show that it coincides with *α* where *ϕ* first drops below 1.

#### 2.1.3 Allowed subgraphs and their dependence on interaction strength

Here we begin to explain the different regimes described in Section 2.1.2, by analyzing properties of the equilibria of the model. In the limit of small migration (λ_*i*_ → 0^+^) some of the species will persist (*N*_*i*_ > 0) and others go locally extinct (*N*_*i*_ = 0 as λ_*i*_ → 0^+^). At an equilibrium, the extinct species must be unable to invade (*dN*_*i*_/*dt* < 0), and the abundances of the persistent species must return to the fixed point if perturbed away from it. These conditions will be referred to as *uninvadability* and *stability*, respectively. The persistent species can be grouped into connected subgraphs of the community graph, see Fig 1A.

We begin in the limit of very large *α*, studied in [8, 22]. Under this very strong competition, the problem of finding equilibria reduces to choosing sets of coexisting species that satisfy two conditions. First, two interacting species cannot both persist (competitive exclusion). The connected subgraphs are thus individual species, see Fig 1A, rightmost illustration. Second, an extinct species cannot invade if and only if it interacts with one or more persistent species. Stability is automatically satisfied, as it involves isolated persistent species. Importantly, in this limit of strong interactions the exact values of *α* do not appear in these two conditions, and so finding an equilibrium point reduces to a discrete, combinatorial problem on the graph, of finding a maximally independent set [8]. In [22], the authors used this insight to calculate the diversity and number of equilibria on Erdős–Rényi graphs (where the pairs of interacting species are chosen independently with some probability).

At lower values of *α* the connected subgraphs are no longer only isolated species, see Fig 1A. These subgraphs must satisfy certain “internal properties” in order for them to appear at a given *α*. As long as all of the neighboring species to the subgraph are extinct, the abundances at a fixed point of the subgraph are determined entirely by interactions within it. These abundances must be positive (a condition known as *feasibility*), and the fixed point must be stable. These conditions depend only on *α*. This allows us to understand much of the behavior by looking at individual subgraphs: each type of subgraph *μ* will have a critical value $\alpha_{c}^{\left(\mu \right)}$, above which it is either unstable or not feasible, and can therefore only appear at an equilibrium of a system in the “allowed” range $\alpha <\alpha_{c}^{\left(\mu \right)}$. (This leaves out a possibility that a graph could switch back and forth between being allowed or not, see Section A in S1 Text). Thus the system is governed by discrete combinatorial conditions, which determine the entire set of possible equilibria of a given system.

Here another important simplification enters. Sparse random graphs, including random-regular graphs and Erdős–Rényi graphs discussed in Sec 2.2, are locally tree-like, meaning that they have only a finite number of short cycles even when *S* is large. For example, in a large random regular graph with *C* = 3 the average number of triangles is 4/3 [34]. Thus, most connected subgraphs of finite size in the network will be trees, i.e., contain no cycles, and properties such as diversity and species abundance distribution that are averages over the entire community can be calculated by only considering trees, and specifically, the critical values $\alpha_{c}^{\left(\mu \right)}$ need to be found only for trees. Examples of connected subgraphs within a local tree neighborhood are shown in Fig B in S1 Text.

The trees can be divided into chains and other trees. We calculated $\alpha_{c}^{\left(\mu \right)}$ for chains analytically, see Section A in S1 Text. For a chain with *n* species,

$$\begin{array}{c} \alpha_{c}^{\left(\mu \right)}\equiv \alpha_{\text{chain}}^{\left(n\right)}=\{\begin{array}{cc} \frac{1}{2\;\text{cos}\left(\frac{\pi }{n+1}\right)} & n\;\text{even}\\ \frac{1}{2} & n\;\text{odd} \end{array} \end{array}$$ (2)

For chains of even length, $\alpha_{\text{chain}}^{\left(n\right)}$ is a decreasing series that converges from above to $\alpha_{\text{chain}}^{\left(\infty \right)}=\frac{1}{2}$, which is also the critical value for all chains of odd length, $\alpha_{\text{chain}}^{\left(n\right)}=\frac{1}{2}$. All other trees have $\alpha_{c}^{\left(\mu \right)}\leq \frac{1}{2}$, with the first ones appearing, coincidentally, exactly at 1/2, as we prove in Section A in S1 Text. Therefore, $\alpha_{c}^{\left(\mu \right)}>\frac{1}{2}$ only for chains of even length, so only they can appear in communities at *α* > 1/2.

In addition to these “intrinsic” considerations about the stability and feasibility of different connected components, uninvadability must also be considered. This is more complex since it depends on how the components fit together, and in principle this could lead to additional jumps in *ϕ*. However, in the *α* > 1/2 region, such jumps seem to be rare if they exist at all, and their size is so small that we have not detected them in simulations. See details in Section B in S1 Text.

This means that for *α* > 1/2, in each range of *α* between the $\left\{\alpha_{c}^{\left(\mu \right)}\right\}$, the allowed types of trees will not change and so essentially the same set of equilibria will exist (since uninvadability does not seem to be important except at the transitions). As *α* is lowered below some $\alpha_{c}^{\left(\mu \right)}$, a new tree abruptly appears, leading to many new possible configurations and thus causing the diversity to jump. While the dynamical simulations used to obtain *ϕ*(*α*) do not necessarily reach all equilibria with the same probability, they clearly show jumps in *ϕ* at these values, with plateaus of approximately constant values of *ϕ* in between. Fig 1B shows the function *ϕ*(*α*), marking some of the critical $\alpha_{\text{chain}}^{\left(n\right)}$ from Eq (2) as dashed vertical lines, showing that the jumps in *ϕ* indeed happen exactly at $\alpha_{\text{chain}}^{\left(n\right)}$. This also happens for trees that are not chains when *α* < 1/2, see an example in Section A in S1 Text.

As *α* is lowered, infinitely many subgraphs of more complex structures become stable, so the values $\left\{\alpha_{c}^{\left(\mu \right)}\right\}$ become more dense, and the jumps in *ϕ*(*α*) smaller (see Fig C in S1 Text). This makes it harder to observe them in numerics, but we expect that they exist in the entire range down to *α*_UE_, defined in the following. Once trees appear there are many interesting types of transitions that could happen. Just as at 1/2 arbitrarily long chains appear, there could be other points where there are qualitative changes in the properties of trees; see the Discussion section for further discussion.

We note that properties of the system, such as the value of *ϕ*, cannot be determined without consideration of dynamics. Indeed, the dynamics of Eq (1) is more likely to reach some equilibria over others. For instance, for an Erdős-Rényi graph with mean degree *C* = 3 and interaction strength *α* > 1, the likeliest relative diversity calculated when assuming all equilibria are equally likely is *ϕ* ≃ 0.427 [22]; yet in dynamical simulations at *α* = 1.1 we find *ϕ* = 0.514±0.003.

**Fig 2:**
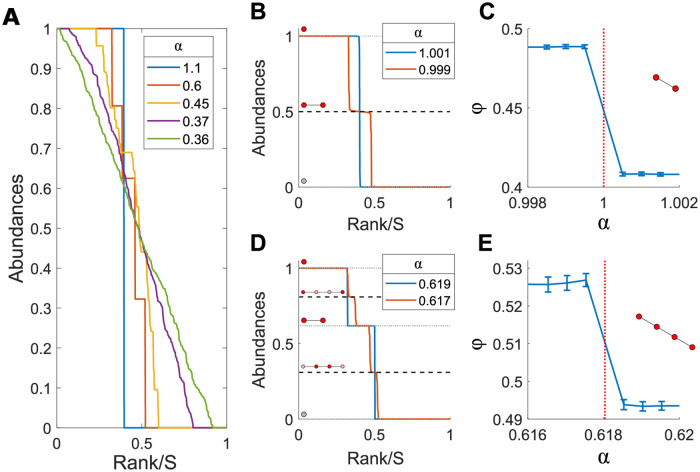
Changes in feasible and stable trees are reflected in species abundances. At each value of the interaction strength *α*, certain trees are allowed, and the abundance of a species depends only on *α* and the position within a tree. (A) The rank-abundance curves at equilibria reached dynamically for *S* = 400, *C* = 3, at several values of *α*. As *α* decreases, the increasing number of feasible and stable trees generates more possible species abundances. (B-E) Relative diversity and species abundances on both sides of two transitions at $\alpha_{\text{chain}}^{\left(n\right)}$ for *n* = 2, 4, where new trees appear. (C) and (E) show the behavior of *ϕ* around the transitions associated with pairs of species and chains of length 4 respectively becoming feasible and stable. (B) and (D) show the abundances at equilibria at values of *α* on two sides of the transitions. The expected abundances are marked by dashed black lines, with thicker lines for the abundances of the species in the tree associated with the transition. Next to each abundance appears the tree that contains it, with the species that have this abundance in dark red (or gray in the case of the abundance 0 of extinct species).

The transitions are also reflected in the possible abundances of species, as seen in rank-abundance curves, which show the abundances sorted in decreasing order, see Fig 2. At a given *α*, the abundance of a species depends only on the connected tree it belongs to, and its position within it; for example, species that belong to a chain of length two have $N_{i}=\frac{1}{1+\alpha }$. Therefore, as a tree *μ* becomes feasible and stable at $\alpha =\alpha_{c}^{\left(\mu \right)}$, the abundances associated with it can appear at an equilibrium. As shown in Fig 2A, this causes the abundance graphs to smooth out as *α* is lowered, since the number of possible abundances increases.

To summarize, in this section we described how the interaction network breaks up into connected subgraphs, with changes in allowed subgraphs driving jumps in diversity and species abundances. These subgraphs are trees that are feasible and stable at that interaction strength. Finding the equilibria of Eq (1) reduces into a discrete graph theoretical problem on the community graph. Broadly speaking, for stronger competition there are fewer and typically smaller allowed trees.

As *α* is lowered, the size of the allowed subgraphs grows until they span a finite fraction of the species, as discussed in the next section. The number of different types of allowed graphs quickly grows with their size, and the problem of classifying them becomes more difficult, and less useful. These very large connected graphs can include the rare but still existing cycles in the graphs, and so they are no longer trees.

#### 2.1.4 Percolation transition

Percolation transitions are one of the canonical phenomena studied in graph theory, and appear in various contexts in community ecology (e.g., [1, 35, 36]). In site percolation, some vertices of a graph are removed. As the probability of vertex removal varies, on one side of the transition the remaining graph breaks into small (sub-extensive) pieces; on the other side, a finite fraction of vertices belong to a single connected component. Natural communities belonging to both regimes are known to exist [1].

**Fig 3:**
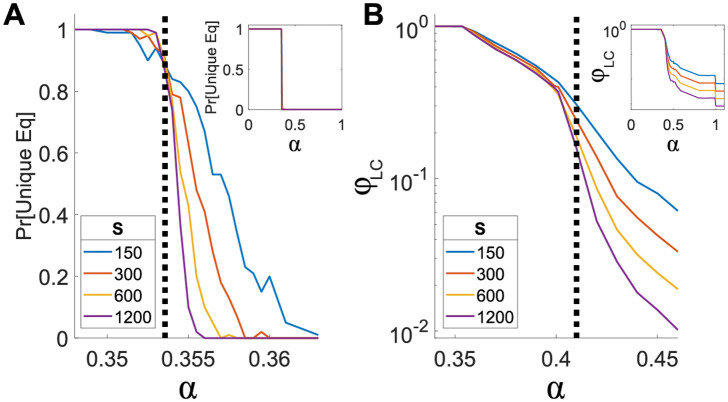
Collective transitions. (A) Unique to multiple equilibria transition: the probability for a unique equilibrium as a function of *α*, for connectivity *C* = 3 and several pool sizes *S*. The probability is obtained by generating many realizations of interaction matrices and determining whether there is a unique equilibrium by the stability of the fully-feasible fixed point, as described in the text body. The exact value for the transition, calculated using Eq (3), is shown as a dashed black line. Inset: the same graph over a larger range of *α*. The transition becomes sharper with *S* grows, as expected from a collective transition. (B) Percolation transition: the fraction of species in the largest connected component as a function of *α*, for several values of *S*. The location of the transition is *α*_perc_ ≈ 0.41 ± 0.01 (dashed black line). At *α* < *α*_perc_ a finite fraction of species belongs to the largest component even when *S* grows. At *α* > *α*_perc_, this fraction decreases with *S*. Inset: the same graph over a larger range of *α*. Here too, the transition becomes sharper at larger values of *S*.

We find that at some interaction strength *α*_perc_ there is a percolation transition, below which the largest connected subgraph formed by surviving species is extensive, that is, includes a finite fraction of all the species. Fig 3B shows the fraction of species belonging to the largest connected component as a function of *α*, for several values *S* with *C* = 3. Above a certain *α*, which for this connectivity is at *α*_perc_(*C* = 3) ≈ 0.41±0.01 (marked by a dashed line), this fraction drops as *S* increases, indicating a sub-extensive largest component. Below *α*_perc_ this fraction converges to a constant value. As expected, this value is smaller than 1/2, since at $\alpha >1/2=\alpha_{\text{chain}}^{\left(\infty \right)}$ the only possible components are finite-length chains, as shown in Sec 2.1.3 above. Also, *α*_perc_ ≥ *α*_UE_ where all species persist, see Sec 2.1.5 below. The fact that the transition becomes sharper with growing *S* is a hallmark of a collective transition.

Fig 3B is qualitatively similar to that of a standard site-percolation transition [37], where vertices are randomly and independently chosen to be “present”. This similarity is used in order to estimate the value of *α*_perc_, as in random regular graphs this is the value in which the relative size of the largest component is proportional to *S*^−1/3^ [38, 39], see further details in Section D in S1 Text. However, the fraction of persistent species at *α*_perc_ is around *ϕ*_perc_ ≈ 0.64 ± 0.02, which is larger than the *ϕ*_perc_ = 1/2 of a standard site percolation transition at *C* = 3 [37]. This is because in our model, the species that persist are not sampled independently; the higher value in our model is expected given that persistent species are correlated, tending not to be adjacent to one another.

The percolation transition marks an abrupt change in the connectivity of the network. Unlike the other transitions discussed here, we have not observed any other sharp changes occurring at this transition, in terms of diversity, stability or other measures beyond the graph connectivity.

#### 2.1.5 Unique to multiple equilibria transition

We find that the final transition in the model with all-equal *α*, at the lowest value of *α*, is from multiple to unique equilibria. In order to find the critical value of *α* for this transition, we will first argue that the community has a unique equilibrium exactly when it is “fully feasible”, i.e. all species are persistent (*ϕ* = 1); if the fully-feasible state is an equilibrium then it is necessarily unique. Thus, the transition from the multiple equilibria phases to the unique equilibrium phase occurs at the value *α*_UE_(*C*) in which *ϕ* drops below 1. The equivalence holds only for this model where all species have the same number of interacting pairs and all interactions have the same strength *α*, and breaks in more general cases, see Sec 2.2 below.

To understand this relation, consider the Lyapunov function *F* = 2∑_*i*_ *N*_*i*_ − ∑_*ij*_ *N*_*i*_*α*_*ij*_*N*_*j*_, which for symmetric interactions (*α*_*ij*_ = *α*_*ji*_) grows with time according to the Lotka-Volterra equations [27], and whose local maxima coincide with the equilibria. The fixed point where all species persist is always feasible, as from the local homogeneity of the community graph all abundances are equal $N_{i}=\frac{1}{1+C\alpha }>0$, and this would be stable if the full interaction matrix *α*_*ij*_ is positive definite. As *α*_*ij*_ is also the matrix of second derivatives of *F*, if the fixed point is stable then the Lyapunov function is concave everywhere, meaning the maximum at the “fully feasible” equilibrium is global and therefore unique.

Conversely, if the fully feasible equilibrium is not stable, then *F* is a non-concave quadratic function on the quadrant {∀*i*: *N*_*i*_ ≥ 0} and one expects that if there are many species, it is likely to have many local maxima, and therefore multiple equilibria. We checked this relation numerically, by generating 100 realizations of the interaction matrix at a given *α*, solving the dynamics in Eq (1) with 30 different randomly chosen initial conditions, and checking whether all runs converge to the same equilibrium. This process was repeated around the transition (whose position is given below in Eq (3)), for *α* ∈ [0.35, 0.36] when *C* = 3 and for *α* ∈ [0.285, 0.3] when *C* = 4, and with *S* = 200, 400. In all runs, there was a unique fixed equilibrium at exactly the same realizations that were fully feasible.

The stability is thus determined by the range in which the matrix *α*_*ij*_ = *δ*_*ij*_ + *αA*_*ij*_ is positive definite. *A*_*ij*_ is an adjacency matrix of a *C*-regular graph of size *S*, and at large *S* its minimal eigenvalue is with probability one at $\lambda_{\text{min}}^{A}=-2\sqrt{C-1}$ [40]. The minimal eigenvalue of the matrix *α*_*ij*_ is therefore at $\lambda_{\text{min}}=1+\alpha \lambda_{\text{min}}^{A}=1-2\alpha \sqrt{C-1}$, and the critical value of *α* will be

$$\begin{array}{c} \alpha_{\text{UE}}(C)=\frac{1}{2\sqrt{C-1}}\;. \end{array}$$ (3)

Fig 3A shows the probability of the system having a unique equilibrium as a function of *α* for several values of *S*, using the stability of the matrix *α*_*ij*_. As *S* increases, the probability for a unique equilibrium becomes sharper (again, a clear sign of a collective transition), approaching a step function at the expected value of the transition *α*_UE_(*C*).

Eq (3) highlights a somewhat surprising difference between a sparse system at large connectivity *C* (the limit *C* → ∞ while keeping *C* ≪ *S*) and a fully interacting system (where *C* = *S* → ∞). As the connectivity *C* grows, *α*_UE_ approaches zero; so the unique equilibrium phase shrinks to a tiny range of *α*. In contrast, as discussed in section 2.1.2, for fully-interacting systems the unique equilibrium phase extends from *α* = 0 to *α* = 1.

### 2.2 Heterogeneity in vertex degree and interaction strength

**Fig 4:**
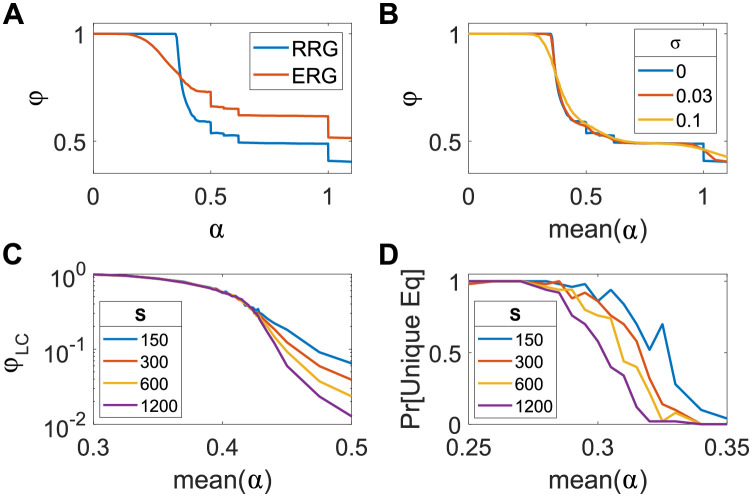
Transitions with heterogeneous interaction strengths and degrees. (A-B) Transitions due to changes in allowed trees are broadened when there is variability in interaction strengths, but remain sharp for variation in degree. The relative diversity *ϕ* as a function of interaction strength *α*, for *S* = 400, *C* = 3. (A) Erdős-Rényi graphs with interaction probability *p* = *C*/*S*, compared to a random *C*-regular graph. (B) Interaction strength is drawn from a normal distribution with mean *α* and standard-deviation *σ* = 0, 0.03, 0.1, keeping the interactions symmetric and a random regular graph. (C-D) The collective transitions with heterogeneity in interaction strength become sharper as *S* increases, just as they do without it (compare with Fig 3). Results are shown for *C* = 3, *σ* = 0.1 and several values of *S*. (C) Percolation transition: The fraction of species in the largest connected component as a function of *α*. (D) Unique to multiple equilibria transition: The probability of having a unique equilibrium as a function of *α*.

So far, Sec 2.1 analyzed a model where each species interacts with exactly *C* others, and all with the same interaction strength *α*. Here we consider the effects of heterogeneity, both in the strength of species interactions and in the vertex degree (the number of species interacting with a given one). The interaction strength is varied by drawing it from a normal distribution with mean *α* and a given standard deviation *σ*. The degree is varied by replacing the random regular graphs with an Erdős-Rényi random graph, in which each pair of species is independently chosen to interact with probability *C*/*S*, such that the average degree is *C*. To understand how these two changes affect the results, we consider them separately. Fig 4 shows the relative diversity *ϕ* as a function of *α* or mean(*α*), for both cases, compared with the random *C*-regular all-equal *α* case (with *C* taken to be the same as the average connectivity of the Erdős-Rényi graph).

When varying the degree, the jumps in the relative diversity *ϕ* due to changes in the allowed trees remain sharp, while they are broadened for variations in interactions strength. This makes sense, as the trees can still exist if the degrees vary; there may be additional adjacent species but these do not affect whether the populations on the tree are feasible and stable. On the other hand, the interaction strengths affect the stability and feasibility of the tree. In an Erdős-Rényi graph without variation in the interaction strength, all trees of the same topology will all have the same *α*_*c*_. (*ϕ* is different between the Erdős-Rényi and regular graphs due to their different structure).

If interaction strengths are varied, trees in the same system which have the same topology but different interaction strengths, might have different limits on stability and feasibility, leading to the appearance of more types of allowed trees than in the all-equal *α* case. But if the disorder is not too strong, the picture of the all-equal *α* case remains relevant: if the mean value of *α* is within a plateau of the all-equal *α* case and not too close to the ends, the interactions would mostly allow the same trees as they would in the case without disorder. For example, for mean(*α*) = 0.7, within the plateau allowing only pairs and singlets, for *σ* = 0.1 these make up 99.5% of feasible trees in a typical equilibrium for large *S*. Indeed, for *σ* = 0.1, in most of the range within this plateau, *ϕ* is almost identical to the all-equal *α* case Fig 4B. However, this picture does not hold in the regime of $\alpha <1/2=\alpha_{\text{chain}}^{\left(\infty \right)}$, where the set of allowed subgraphs is much more sensitive to the heterogeneity. In the all-equal *α* case, the critical values $\left\{\alpha_{c}^{\left(\mu \right)}\right\}$ are much more dense in this range than for the short chains at $\alpha >\alpha_{\text{chain}}^{\left(\infty \right)}$ (see Section A in S1 Text). Therefore, for a given value of *σ* at some $\alpha <\alpha_{\text{chain}}^{\left(\infty \right)}$, subgraphs of many more different shapes would become allowed or disallowed as a result of the heterogeneity than for the same value of *σ* in most of the range $\alpha >\alpha_{\text{chain}}^{\left(\infty \right)}$.

As a rule of thumb, the width of the broadened regions replacing the jumps will be proportional to the width *σ* of the distribution of *α*. The precise shape of these broadened regions would have some dependence on the distribution of *α* (e.g., whether it is Gaussian, uniform or otherwise). This is in contrast to fully interacting networks, where only low moments of the distribution of *α* affect the community properties [19].

The two remaining transitions, for percolation and from unique to multiple equilibria, both appear to become sharper as *S* increases for both variations in interaction strengths and degree, as can be seen in Fig 4C and 4D and in Fig F in S1 Text. For any given *S* the transitions are broader compared to the equal-*α* model (Fig 3). More work is needed to understand the properties of this transition and its location (we note that an analogous transition in a fully-interacting system, known to be asymptotically sharp, is similarly broadened at finite *S* [12]).

Furthermore, in both cases *ϕ* drops below 1 while still at the unique equilibrium phase, which happens when the system is no longer fully feasible. This is shown for both Erdős-Rényi random graph and for varying interaction strengths in Fig F in S1 Text. This is in contrast to the all-equal *α* model, where *ϕ* drops below 1 when the system becomes unstable at *α*_UE_.

### 2.3 Subgraph emergence rule: How the trees grow

As the interaction strength is lowered (by lowering *α* if it is constant, as in Sec 2.1.1 or changing mean(*α*) when interactions are heterogeneous, as in Sec 2.2), the allowed connected subgraphs become larger (containing more species) and more complicated, until one connected subgraph can take up a finite fraction of the community at the percolation transition. As the interaction strength keeps decreasing, this extensive connected subgraph continues to grow until it finally includes the entire network. For *α* > 1/2 there is a clear regularity in the sequence of transitions, as even-length chains become allowed by order of length. This raises the question of whether there is any regularity by which more complicated subgraphs (trees, and even subgraphs with cycles) become allowed. We now describe a general and simple result, when the interaction strengths are heterogeneous.

**Fig 5:**
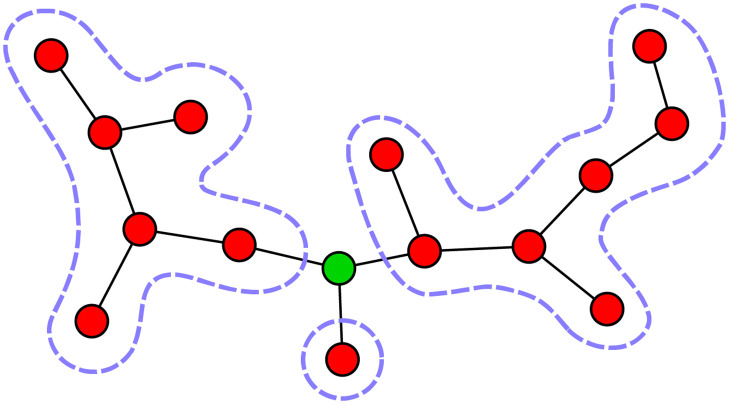
A tree that becomes feasible and stable at some *α*_*c*_, can be constructed from three or more trees that are allowed right above *α*_*c*_ (surrounded by dashed lines), joined by one additional species (green). This is true with probability one when there is heterogeneity in interaction strengths.

Consider a subgraph within the interaction network, see Fig 5. Since interaction strengths are not all equal, this refers to a specific set of vertices, which means the result can be different for the same subgraph structure when it involves different species. To define *α*_*c*_ of the subgraph, consider the process by which the mean strength is changed by shifting the values of the *α*_*ij*_, i.e. adding a constant (other continuous changes of the matrix *α* are also possible). Just below *α*_*c*_ all abundances are positive. We prove in Section E in S1 Text that with probability one, it is feasibility, rather than stability, that is lost at *α*_*c*_, by one species going extinct, with *N*_*i*_ vanishing continuously as *α* → *α*_*c*_. (This relates to results on other systems showing that if the entire system is feasible then generically it would also be stable [41–44]). When this species becomes extinct, the remaining subgraph splits up into allowed subgraphs. This means that there is a hierarchical relationship, where the allowed subgraph right below *α*_*c*_ is composed of subgraphs allowed right above *α*_*c*_, with one additional vertex.

For a tree subgraph, the species that goes extinct interacts with at least three species in that subgraph (meaning that it is a branching point), assuming that the distribution of the *α*_*ij*_-values is not too wide. See argument in Section E in S1 Text. This implies that the tree splits into three or more trees.

This construction gives a constraint on what order the specific subgraphs become allowed, i.e. become feasible and stable: that when a subgraph becomes allowed as *α* is decreased, pieces of it with one species removed were already allowed. As noted above, because of the heterogeneity of the interactions, here a subgraph refers to a specific set of species on which it resides. Note that as always, whether an allowed graph appears in an equilibrium depends also on the neighboring species and the rest of the network.

## 3 Discussion

We have looked at a community assembled from a pool of sparsely-interacting species. When the interactions are strong enough, the assembly process breaks the network into many connected subgraphs. The problem of equilibrium coexistence reduces to understanding which subgraphs are allowed, and how they are organized to keep extinct species from invading.

When these subgraphs are small, it might be possible to formulate predictive local rules about their occurrence, in the spirit of “assembly rules” [4, 45, 46]. The simplest example is competitive exclusion, where if the interactions between two species are greater than one, *α*_*ij*_, *α*_*ji*_ > 1, then they cannot coexist within a community of species that interact competitively, irrespective of the state of the other species. This can be interpreted as a rule that when the interaction strength is stronger than one, the connected components include just one species. Here this regularity extends to weaker interaction strengths, first identifying a regime where interacting pairs are also allowed, which is quite robust to some level of heterogeneity in interactions strengths (Sec 2.2), and then to regimes with larger connected subgraphs.

For lower interaction strengths there are many larger allowed subgraphs, making the corresponding graph-theoretical problem hard and far less local, and limiting the potential for predictive local rules. At even lower interaction strengths, connections percolate across the entire network of coexisting species, and below that there is a dramatic transition in behavior, as the equilibrium becomes unique, similar to transitions found in fully-interacting systems [12, 23, 24]. We have not observed any sharp changes occurring at the percolation transition, to diversity, stability or other measures beyond the graph connectivity; percolation might however be a necessary bridge between the finite-subgraph regime, and the unique to multiple-equilibria transition.

Communities where the community graph percolates, as well as ones consisting of small disconnected subgraphs, are known to exist in nature [1]. It would be interesting to experimentally test our prediction, that the community breaks down into smaller subgraphs as interaction strengths increase. This would require control of interaction strengths (for example as in [47]). In the same way the prediction of the hierarchy of subgraph growth in section 2.3 could be tested.

In a striking difference from fully-connected networks, the rich phenomenology we found is present even when the interactions strengths are all the same, as are the number of competitors per species; thus, while the interactions are locally ordered, with the neighborhoods of almost all species being identical, abundances can vary greatly. This is in contrast with fully-interacting networks, where a variability in interaction strength is necessary for non-trivial phenomena to occur, and has been central to much of the field for decades [8, 11–17]. This makes the interaction strength (or its mean as opposed to the width of the distribution) a parameter of independent importance, single-handedly driving changes in stability and feasibility. When heterogeneity is present, the mean and distribution of interaction strengths have a combined effect, with the allowed subgraphs still playing a central role in shaping the community.

There are also many mathematical questions to explore in these systems, which are interesting because of the interplay between the combinatorial structure of the community graphs and the quantitative properties of the interaction matrices. Such questions include a further understanding of the sequence of transitions: Are there other limit points where infinite trees become stable (such as the infinite chain becoming stable at *α* = 1/2)? And are there ranges where the critical points $\alpha_{c}^{\left(\mu \right)}$ are dense? It would be especially interesting to understand the transition to the unique equilibrium state, by studying the structure of the small groups of species that go extinct just above the transition. It would likely be possible to make progress on many of these questions by studying ideal infinite trees with a fixed degree *C*, so that the inhomogeneity in the equilibria arises from their instabilities.

Another question is how much the dynamics affects the distribution of equilibria reached, which is not uniform over all possible equilibria, as discussed in Section 2.1.3, and how much this affects the results, such as the sizes of the jumps in diversity. The effect of noise, not considered in this paper, is also very interesting. The transient dynamics when relaxing towards equilibria can be of interest in itself, particularly if long periods of time are spent near unstable fixed points on the way to a stable one. Finally, going beyond the models discussed in this paper, when interactions are asymmetric the dynamics might never reach an equilibrium, opening a wide field for further research.

The extent to which interactions in different natural communities are sparse is an open question, since directly measuring interaction strengths can be hard, especially the weaker ones. This is complicated by additional factors, as many weak interactions might have a large cumulative effect, and that some inference techniques assume that the network is sparse (e.g., [48]). One can hope that studying consequences of sparsity would help identify and better understand such communities.

## Supporting information

S1 Text Appendices. Details of theory and simulations.
(PDF)
