# Supplementary material for: Local and collective transitions in sparsely-interacting ecological communities
Source: PLoS Comput Biol. 2022 Jul 11;18(7):e1010274. doi: 10.1371/journal.pcbi.1010274 (PMC9302738; doi:10.1371/journal.pcbi.1010274)
Supplement: S1 Text — Details of theory and simulations. (PDF) [file pcbi.1010274.s001.pdf]

Supporting Information for:
Local and collective transitions in sparsely-interacting ecological communities

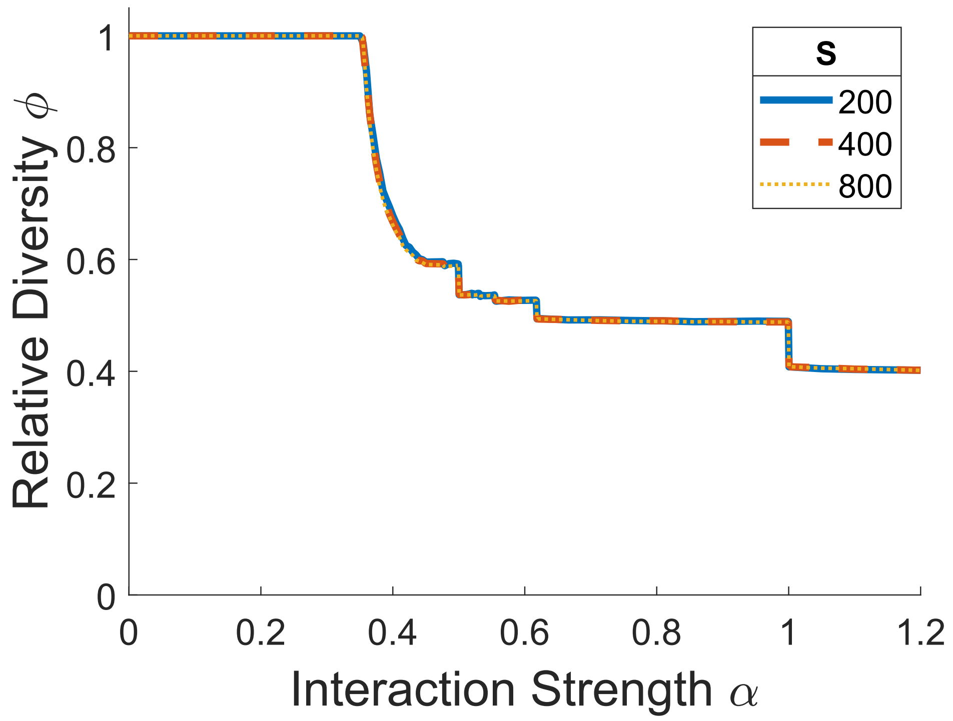

Figure A: **The behavior of the relative diversity is convergent with system size S.** The dependence of the relative diversity $\phi = S^*/S$ (where S is the total number of species and S^* the number of persistent species) at equilibrium on interaction strength α, from simulations with sparse interactions with degree $C = 3$, and pool sizes $S = 200$ (blue), $S = 400$ (red, dashed) and $S = 800$ (yellow, dotted). The behavior has clearly converged to that of the limit $S \to \infty$ already at $S = 200$. Importantly, the size of the jumps in ϕ remain finite at infinite pool size.

A Critical values of trees

The critical α values for connected trees, $\left\{\alpha_c^{(\mu)}\right\}$, are discussed in Sec 2.1.3 of the main text. They are the values above which each tree becomes either unstable or unfeasible, and therefore cannot appear in an equilibrium. In rare cases, a stable tree can regain unfeasibility after losing it (see below); here we define $\alpha_c^{(\mu)}$ more precisely as the *highest* value of α where the tree is feasible and stable. As discussed in the main text, trees are important subgraphs because the local neighborhoods of most species are locally tree-like. Examples of trees appearing in an equilibrium in the neighborhood of one species within a large community are shown in Fig B.

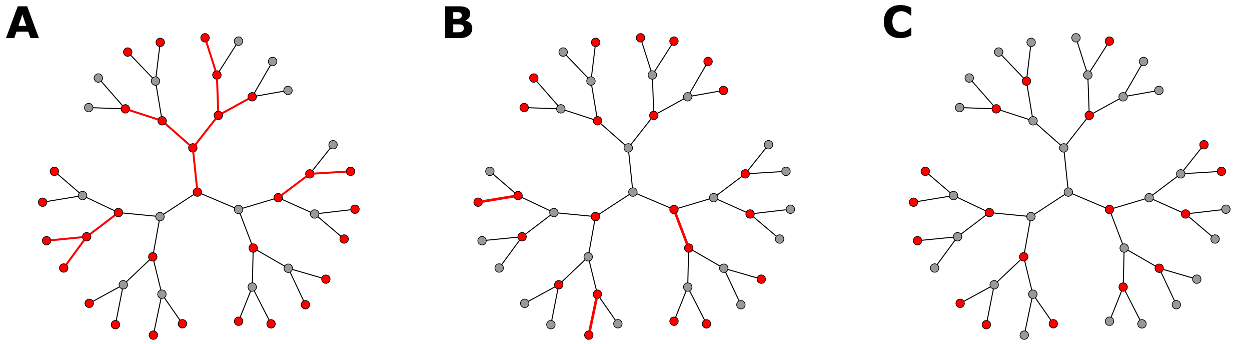

Figure B: **Equilibrium in the local neighborhood of one species.** The neighborhood of one species in a specific system with connectivity $C = 3$ and pool size $S = 1200$, where the interaction strengths are **(A)** $\alpha = 0.45$ **(B)** $\alpha = 0.7$ **(C)** $\alpha = 1.1$. Extinct species are in gray, and persistent species in red. The edges connecting two persistent species are also marked in red. As the interaction strength is increased, the subgraphs change from large trees that are not chains in (A), to length-2 chains and singlets in (B), and singlets only in (C).

Stability changes at a single value of α, so that the system is stable for all values of α below it and unstable for all values above it. Indeed, as the interaction parameters, in matrix form, are represented by $I + \alpha A$, where I is the identity matrix (see Sec 2.1.1 In the main text), if the minimal eigenvalue of the adjacency matrix A representing a tree is λ^A_{min}, the smallest eigenvalue of the tree at the interaction strength α would be $\lambda_{min} = 1 + \alpha\lambda^A_{min}$, so the tree is unstable exactly for $\alpha > -1/\lambda^A_{min}$. Feasibility on the other hand can be gained and then lost more than once, but we find that most stable trees that gain feasibility as α is lowered usually retain it, with feasibility gained and lost again in only 0.03% of trees up to 20 vertices for $C = 3$ and 0.01% of trees up to 15 vertices for $C = 5$.

As mentioned in Sec 2.1.3, we find that for a chain of length n,

$$\alpha^{(n)}_{\text{chain}} = \begin{cases} \frac{1}{2\cos\left(\frac{\pi}{n+1}\right)} & n \text{ even} \\ \frac{1}{2} & n \text{ odd} \end{cases} \tag{1}$$

and for all other trees, $\alpha_c^{(\mu)} \leq \frac{1}{2}$, with μ going over all trees. A histogram of the values for small trees, calculated numerically, appear in Fig C(A), and the example in Fig C(B) shows that they do indeed generate jumps in ϕ even in the $\alpha < 1/2$ region.

While it is fairly easy to determine the critical α's for chains by checking stability and feasibility directly, it is hard to show that all trees that are not chains are either unfeasible or unstable above 1/2. E.g., to show they are unfeasible, we have to find the solutions to the equilibrium equations and check that one of the species has a negative abundance, but there is no general formula for inverting the interaction matrix on an arbitrary tree. Instead of doing this, we can note that if the tree is unfeasible or unstable, it will still have a stable equilibrium (because there is a Lyapunov function, so it cannot oscillate indefinitely), but this stable equilibrium has missing species. It must decompose into subgraphs that are stable and feasible, which are expected to be only chains above 1/2. Conversely, the reasoning from Sec 2.1.5 of the main text shows that if such an uninvadable equilibrium made up of chains exists, the full graph cannot be stable and feasible. This does not require inverting the matrices for trees, just doing a more combinatorial problem of splitting the tree into pieces. This idea and another lemma are the basis for the proof.

These two lemmas, proved in Sec. A.1, are (1) A tree with an unstable sub-tree is itself unstable, and (2) Any subgraph is feasible and stable if and only if there is no stable and uninvadable equilibrium on the tree where some of the species are extinct. We then prove the results on chains, Eq. (1) in Sec. A.2. Although this can be done

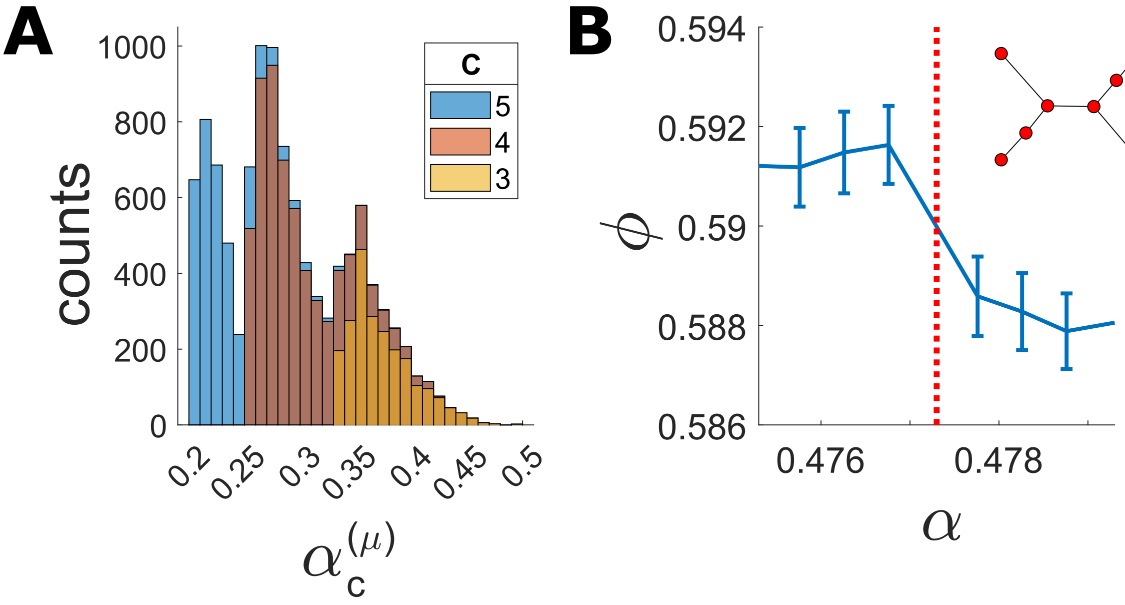

Figure C: **The critical values $\left\{\alpha_c^{(\mu)}\right\}$ for trees that are not chains (A)** Histogram of the critical values for trees up to size 15, excluding chains, which are possible subgraphs of random regular graphs with connectivity $C = 3, 4, 5$. **(B)** Trees can generate jumps in ϕ at $\alpha < 1/2$: behavior of ϕ for $S = 400, C = 3$ around the value $\alpha_c^{(\mu)}$ associated with the tree shown.

directly, the lemmas give more intuitive derivations of parts of the results. For example, Lemma 2 allows us to show that an odd length chain is never feasible and stable above 1/2 by finding an equilibrium with extinct species in that range. We prove in Sec. A.3 that for trees that are not chains $\alpha_c^{(\mu)} \leq 1/2$, in two stages: first, we use lemma 1 to show that unless the junctions in the tree have at least one neighbor of degree 1 and the rest of degree 2 at most, the tree is unstable. Next we prove that, for any junction with these properties, an equilibrium exists for $\alpha > 1/2$ in which the species at the junction is extinct, so by lemma 2, the tree is not feasible and stable.

A.1 Supporting Lemmas

Here we will introduce two lemmas to aid the proof. They are also of interest in their own right.

Lemma (1): A graph that has an unstable subgraph is itself unstable.

Proof: To prove this, denote the interaction matrices of the entire graph and the subgraph as α_{ij} and α^*_{ij} respectively, and their minimal eigenvalues as $\lambda_{\min}, \lambda^*_{\min}$, with $\lambda^*_{\min} < 0$ as the subgraph is unstable. As α^*_{ij} is a principal submatrix of α_{ij}, from the Cauchy eigenvalue interlacing inequality [1] $\lambda_{\min} \leq \lambda^*_{\min} < 0$, meaning the full graph is unstable.

Lemma (2): A graph is feasible and stable if and only if there is no stable equilibrium where some of the species are extinct, that is also uninvadable on the graph.

Proof: If the graph is feasible and stable, then the interaction matrix for the tree, α_{ij}, is positive definite so the Lyapunov function discussed in Sec 2.1.5 of the main text is concave [2] and the equilibrium is unique; hence there is no other equilibrium in which some species is extinct. If it is not feasible and stable, the existence of a Lyapunov function still implies that there must be some equilibrium, and since the graph is not feasible and stable some species in this equilibrium must be extinct.

A.2 Chains

From Lemma 1 we immediately see that the only allowed subgraph at $\alpha > 1$ is a singlet, as any other subgraph includes a length-2 chain, which is unstable in this range.

In order to calculate the $\alpha^{(n)}_{\text{chain}}$, we first derive a rule relating the range of stability and feasibility to the degrees of the vertices of a graph. Using Lemma 2 we show that for any subgraph μ, $\alpha_c^{(\mu)} \geq 1/\left(\max_j C_j\right)$, where C_j is the number of interacting neighbors of species j. For a chain, $\max_j C_j = 2$, and so for any $n \geq 2$, $\alpha^{(n)}_{\text{chain}} \geq \frac{1}{2}$.

Indeed, assume that species j is extinct at a fixed point. Its growth rate is $g_j = 1 - \alpha \sum_{k\sim j} N_k \geq 1 - \alpha C_j$. For $\alpha < 1/C_j$ the growth rate would be positive and the equilibrium would be invadable, so in this range species j cannot be extinct at an equilibrium. So at $\alpha < \min_j (1/C_j)$, no species can be extinct at a fixed point, and at the equilibrium all species persist. This behavior is apparent in the histogram of values $\alpha_c^{(i)}$ calculated numerically in Fig C(A)

For even length chains, $\alpha_c = \left[2\cos\left(\frac{\pi}{n+1}\right)\right]^{-1}$**:** This can be proved by directly checking the feasibility and stability of the chains above 1/2. The interaction matrix representing a chain of length n is an $n \times n$ tridiagonal Toeplitz matrix with 1 on the main diagonal and α on the diagonals above and below. In this case, the kth eigenvalue, with $k = 1, ..., n$, is

$$\lambda_k = 1 - 2\alpha \cos\left(\frac{k\pi}{n+1}\right) \leq \lambda_1 \tag{2}$$

and the chain will be stable for

$$\alpha < \frac{1}{2\cos\left(\frac{\pi}{n+1}\right)} \tag{3}$$

[3]. The chain is also feasible in this range if the abundances are all positive. These abundances equal the sum of the columns of the inverse matrix α_{ij}^{-1}. Denoting its components as σ_{jk}, for $j \leq k$ they are given by

$$\sigma_{jk}^n = (-1)^{j+k} \frac{1}{\alpha} \frac{U_{j-1}\left(\frac{1}{2\alpha}\right) U_{n-k}\left(\frac{1}{2\alpha}\right)}{U_n\left(\frac{1}{2\alpha}\right)} \ , \tag{4}$$

where $U_m(x)$ is the m-th Chebyshev polynomials of the second kind. The sum over the columns reduces to only two entries of the inverse matrix, so the abundance of the k-th species on a chain of length n is [4]

$$N_{n,k} = \frac{1 + \alpha\left(\sigma_{1k}^n + \sigma_{1,n-k+1}^n\right)}{1 + 2\alpha} \ , \tag{5}$$

From Lemma 2 we already obtained that $\alpha_{\text{chain}}^{(2m)} > \frac{1}{2}$, and in the region $\alpha > 1/2$, the nth Chebyshev polynomial would be $U_n\left(\frac{1}{2\alpha}\right) = \frac{\sin((n+1)\theta)}{\sin\theta}$, where $\cos\theta = \frac{1}{2\alpha}$. In the region where the chain is stable, $\alpha < 1/2\cos\left(\frac{\pi}{n+1}\right)$, it is straightforward to show that all abundances are positive.

For odd length chains, $\alpha_c = 1/2$**:** For $\alpha > 1/2$, a chain of odd length has an uninvadable equilibrium where species alternate between persistent and extinct, with the persistent species at the odd positions (See Fig D(A)). The persistent species would have only extinct neighbors and therefore would be stable with abundances $N_i = 1$. Each extinct species would have two persistent neighbors and its growth rate will be negative, $g = 1 - 2\alpha < 0$.

A.3 Trees that are not chains have $\alpha_c \leq 1/2$

To show that such a tree is not allowed above 1/2, we would like to use lemma 2 – if we can remove vertices such that the tree breaks up into chains and the removed vertices are kept from invading by the interactions with the neighbors, we will know that the tree does not also have a fully populated stable fixed point. It is natural to try to remove the species at a junction between three or more branches. We introduce a certain property of junctions and show that if a junction has this property, then when the species at the junction is removed, at equilibrium the abundances of its neighbors are large enough to keep it from invading. If the junction does not have this property, the tree is unstable and therefore if allowed to evolve, some species will naturally become extinct.

Trees with unstable subtrees: There are two specific trees, shown in Fig D(B) and D(C), that are unstable exactly for $\alpha > 1/2$, as can be directly verified. The tree in D(C) is a subtree of all trees that have two neighboring junctions, and therefore all such trees have $\alpha_c \leq 1/2$. Trees that have no neighboring junctions will still have at least one junction (as otherwise they would be chains). For such a tree, if all neighbors of the vertices at the junctions have degree 2, then the tree D(B) is a subtree and it is also unstable at $\alpha \geq 1/2$. It remains to be shown that trees that are not chains and do not contain these two subtrees also have $\alpha_c \geq 1/2$. These trees must contain at least one junction, as they are not chains; vertices neighboring the junctions can have no more than one other neighbor, otherwise the tree contains the subtree in Fig D(C); and at least one neighbor of each junction must have no other neighbors, otherwise the tree contains the subtree in Fig D(B). A visualization of such trees, in the case where the junction has 3 neighbors, is shown in Fig D(D).

All trees that are not chains have $\alpha_c \leq 1/2$**:** As we already know that for $\alpha > 1$ all trees are unstable, let us look at trees for some given $1/2 < \alpha < 1$, and show that they cannot be stable and feasible at this α. We will

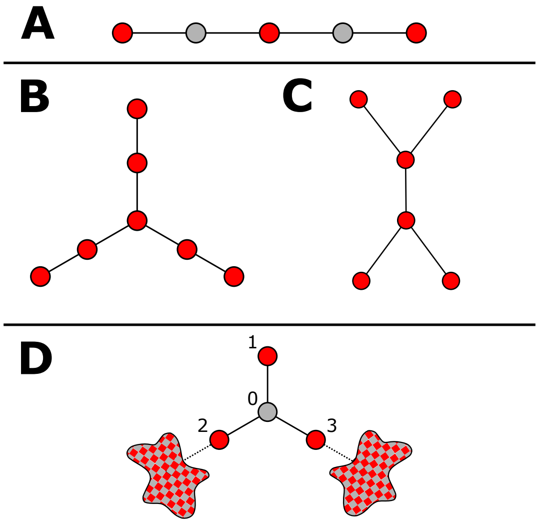

Figure D: **(A)** The equilibrium at $\alpha \geq 1/2$ for odd length chains includes alternating persistent (red) and extinct (gray) species, with the persistent species at the odd positions. **(B-C)** Trees which become unstable for $\alpha > 1/2$. All trees that have these as subtrees are also unstable at this range. **(D)** An equilibrium at $\alpha > 1/2$ for all trees that do not have (B-C) as subtrees. Species 0 as defined in the text, at the middle of the junction, is extinct. It breaks the tree into several smaller subtrees of unknown topology, with its neighbors, species $1, ..., m$ in the text, persistent leaves of each subtree. Specifically, species 1 has no neighbors besides species 0 and is persistent with $N_i = 1$.

also use the fact that for $\alpha < 1$, the leaves of a tree are always persistent at an equilibrium, as a leaf has only a single neighbor, and therefore if leaf i is extinct its growth rate is $g_i = 1 - \sum_j \alpha_{ij} N_j \geq 1 - \alpha > 0$. Further, its abundance is bounded from below, $N_i(\alpha) = 1 - \alpha \sum_{j \sim i} N_j \geq 1 - \alpha$.

If a tree has either of the trees in Fig D(B) and DC as subtrees, then we already showed that it is unstable at α. Otherwise, as explained at the end of the previous part, this tree must have a junction with no neighboring junctions, and at least one neighbor with degree 1, as shown in Fig DD. We now find an equilibrium with extinct species for trees with such a topology.

Mark the species in the middle of the junction as species 0, and its neighbor that has degree 1 as species 1. Species 0 has additional neighbors $2, ..., m$, with $m \geq 3$, as indicated in Fig D(D). Each of these species has at most one more neighbor besides species 0, otherwise there would be a neighboring junction to species 0.

We now show that an equilibrium exists where species 0 is extinct. As species 0 goes extinct, the tree separates into m distinct subtrees of size $< N$. Let us examine the equilibria of these subtrees. The first subtree includes only species 1 (as it had no other neighbor besides species 0), so it has an equilibrium where species 1 is persistent with $N_1 = 1$. All other subtrees also have an equilibrium, because of the existence of a Lyapunov function for each subtree separately. We now need only check that species 0 cannot invade at this equilibrium.

Specifically, as species $2, ..., m$ are leaves of their respective trees, they must be persistent at these equilibria, and have abundances $N_i(\alpha) \geq 1 - \alpha$. The growth rate of species 0 is therefore

$$g_0 = 1 - \alpha \sum_{i=1}^{m} N_i \leq 1 - \alpha - \alpha \sum_{i=2}^{m} (1 - \alpha)$$
$$\leq 1 - \alpha (1 + 2(1 - \alpha)) \leq 0$$

and indeed it cannot invade. So the tree has an equilibrium with extinct species, and thus it cannot be stable and feasible.

B Effects of invadability and non-tree subgraphs

In the main text, we explain that the jumps in the relative diversity $\phi(\alpha)$ in the region $\alpha > 1/2$ result from changes in the stability and feasibility of trees. We neglect the effect of changes in the feasibility and stability of subgraphs that are not trees, and the invadability of extinct species. In this section we will discuss these assumptions and show that such changes do not appear to generate additional jumps in ϕ.

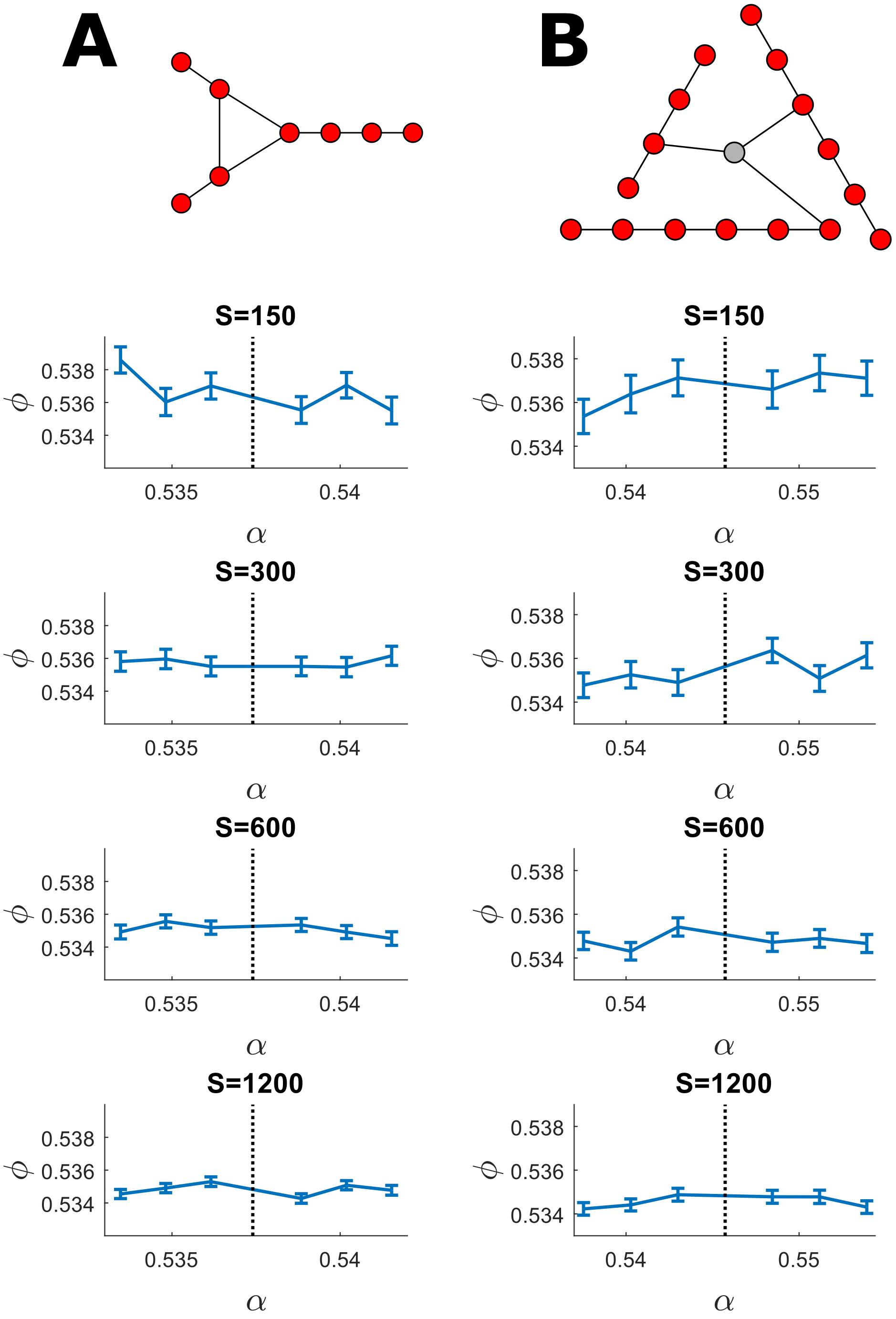

Figure E: **Non-tree subgraphs and invadability changes do not generate jumps in ϕ at $\alpha > 1/2$.** The dependence of relative diversity ϕ on the interaction strength α for increasing pool sizes S, in two cases **(A)** Around $\alpha \approx 0.537$, the value at which a non-tree subgraph becomes allowed. The subgraph is shown at the top **(B)** Around $\alpha \approx 0.546$, the value where an extinct species (in gray) with a set of persistent neighbors as shown will change the sign of its growth rate, and so its invadability.

B.1 Invadability

In this section, we will show that for $\alpha > 1/2$, most extinct species would not change their invadability within the ranges where there is no change in feasibility and stability of trees. In the cases where they do, the jumps generated, if they exist, are so small that we do not observe them in our simulations

A change in invadability occurs at α-values where there is a sign change of the growth rate of an extinct species, $g_i(\alpha) = 1 - \alpha \sum_{j \sim i} N_j(\alpha)$. For $\alpha > 1/2$ the only allowed trees are even length chains, with a finite number of possible abundances $N_j(\alpha)$, and so a finite number of possible growth rates $g_i(\alpha)$, depending on the different possible combinations of neighboring species. For example, in the range where the only allowed trees are singlets and length 2 chains, the only possible abundances are $N_i(\alpha) \in \left\{0, 1, \frac{1}{1+\alpha}\right\}$ and for a C-regular graph, this gives $\begin{pmatrix} C+2 \\ C \end{pmatrix}$ possible growth rates. We checked for such sign changes for $C = 3$ in the range that allows chains of up to length 2,4,6 and 8, about $\alpha \in [0.52, 1]$. We exclude the values $\alpha_{\text{chain}}^{(2n)}$ where we already know there are jumps in ϕ due to changes in chain stability. We stop at the maximal chain length of 8 as the number of possible growth rates grows very fast with the maximal allowed chain length. In the ranges allowing chains of up to length 2 and 4, no growth rate changes sign; in the range allowing chains of up to length 6, one possible growth rate out of 120 changes sign; in the range allowing chains of up to length 8, 5 out of 364 possible growth rates change sign.

In Fig E(B), we show $\phi(\alpha)$ around a value of α where one of these changes in growth rates occur, along with the specific combination of neighbors that generates the change. Even with an increase in the pool size S, we see no jump in the value of ϕ within statistical error. These results could be expected, as each growth rate occurs only when an extinct species has neighboring chains of very specific lengths , which happens very infrequently. However, this does not mean that invadability is unimportant, as it drives the changes in the allowed subgraphs. For example, chains of length 4 become allowed at the value of α such that an extinct species which neighbors a length two chain (of persistent species) and another single species can invade, so that all the sites stick together as a length 4 chain. This is just another way of describing the result above, that a graph is not allowed if removing some species from it leads to a subgraph such that the removed species cannot invade.

B.2 Non-tree subgraphs

As mentioned in the main text, as sparse graphs are tree-like and short cycles are rare, we expect to see no jumps generated by subgraphs that are not trees. Fig E(A) shows an example for a specific subgraph that includes a cycle: $\phi(\alpha)$ displays no jump around the critical value where this subgraph becomes allowed, even as we increase S.

C Collective transitions with heterogeneity

In this section we continue to examine the two collective transitions, the transition from multiple to unique equilibria and the percolation transition, in cases where interaction strengths and vertex degrees are not constant across the network. The behavior at the transitions is shown in Fig 4 in the main text for heterogeneous interaction strengths, and here in the top panels of Fig F for variability in vertex degree modeled by an Erdős-Rényi graph. For both cases, the transition from multiple to unique equilibria becomes sharper as S increases (within the range checked numerically), with the probability of a unique equilibrium approaching a step function. The percolation transition in both cases is qualitatively similar to the transition that occurs in the case with no heterogeneity, as well as to standard site percolation, see section D.

Fig F(C) and F(D) shows that for both types of heterogeneity, ϕ drops below 1 before the transition to multiple equilibria, for $\alpha < \alpha_{\text{UE}}$. Therefore, the feasibility of the entire system is lost before its stability. For heterogeneous interaction strengths, this follows from Lemma (3) in Appendix E.

D Comparison to standard percolation

Here we elaborate on the comparison in the main text between percolation in our model and standard site percolation. In standard percolation, each vertex is taken to be "present" with a given probability p, and for C-regular graphs the percolation transition is known to occur at $p_{\text{perc}}(C) = \frac{1}{C-1}$ [5]. Fig G compares three cases: standard percolation on a random regular graph, percolation in the equal-α model where interaction strength and degree are constant, and for heterogeneous interaction strengths. For each we show the dependence of the fraction of species in the largest connected component, ϕ_{LC}, on the fraction of surviving species, ϕ, or on p for standard percolation.

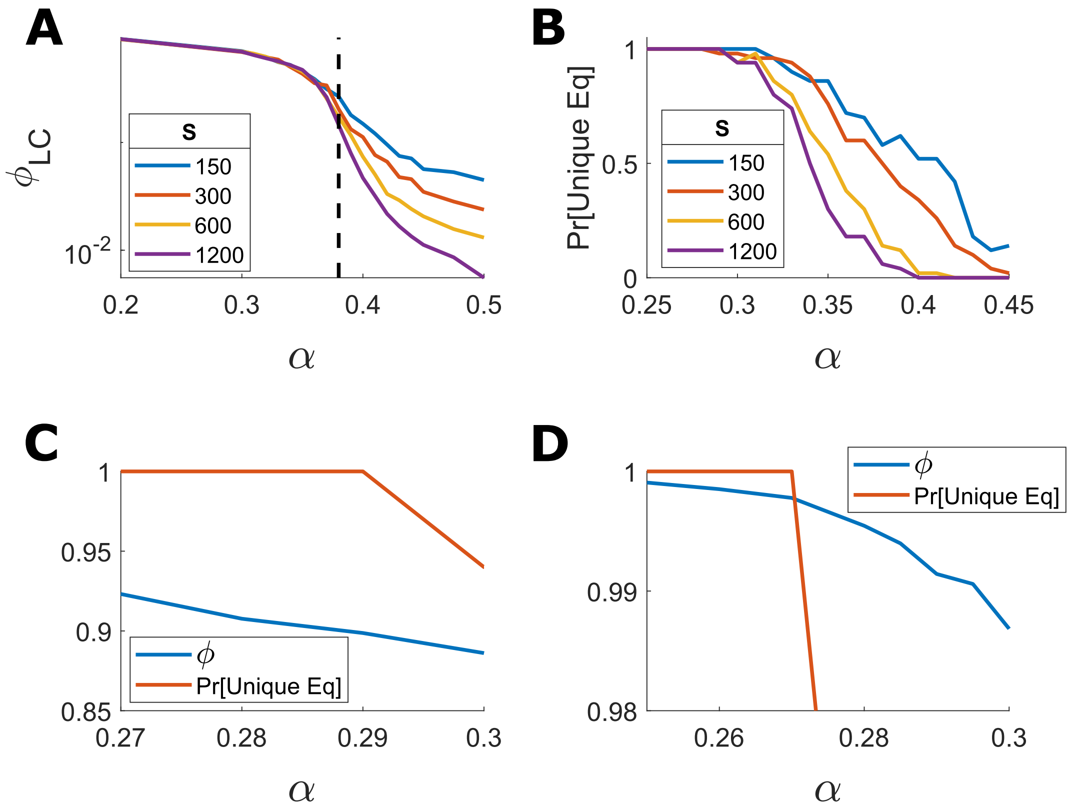

Figure F: **Collective transitions with heterogeneity.** Results in panels (A-B) are the equivalent of Fig 4C and 4D of the main text, here for heterogeneity in degree the transitions. As in Fig 4C and 4D, the transitions become sharper as S increases, within the range of S tested numerically. They show simulation results for Erdős-Rényi graphs with average degree $C = 3$ and several values of S. **(A)** Percolation transition: The fraction of species in the largest connected component as a function of α. At low values of α a finite fraction of species belong to the largest component, and above a transition the fraction of species decreases with S. **(B)** Multiple to unique equilibrium transition: The probability of having a unique equilibrium as a function of α. **(C-D)** For both types of heterogeneity some species go extinct (a loss of feasibility of the entire system) before loss of stability: The fraction of surviving species ϕ drops below 1 in the unique equilibrium phase. Results are shown for $S = 1200, C = 3$. The probability for a unique equilibrium is shown in red, and ϕ in blue. **(C)** Heterogeneity in degree, Erdős-Rényi graphs **(D)** Heterogeneity in interaction strength, $\sigma = 0.1$.

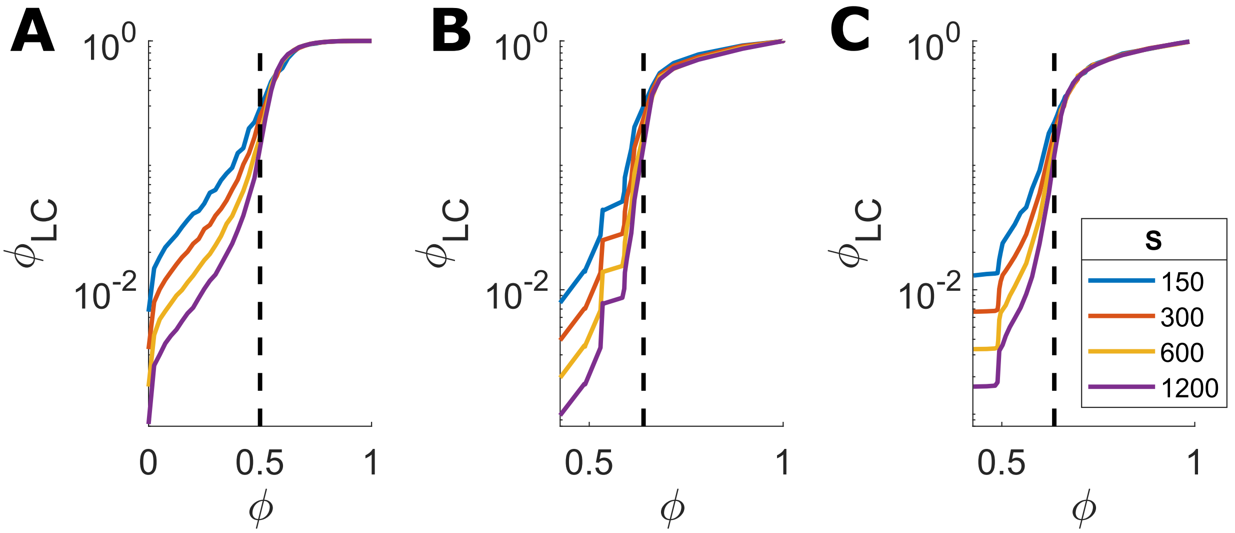

Figure G: **Percolation in our model is qualitatively similar to standard site-percolation, near the transition.** The dependence of the fraction of species in the largest connected component on the fraction of all persistent species ϕ, for $C = 3$ and several pool sizes S. **(A)** Standard site-percolation: vertices are taken to be present with probability ϕ, independently for each vertex. The percolation transition occurs at $\phi_{\text{perc}} = 1/2$, where $\phi_{LC} \sim S^{-1/3}$. **(B)** The equal-α model, with constant interaction strength and vertex degree, $\phi_{\text{perc}} \approx 0.64$. **(C)** The model with variability $\sigma = 0.1$ in interaction strength, $\phi_{\text{perc}} \approx 0.636$.

As mentioned in the main text, in all cases the behavior close to the transition is qualitatively similar. We use this similarity to estimate α_{perc} in our model, as the value of α where the fraction of species in the largest component grows as $S^{-1/3}$, as in known to occur at p_{perc} for standard site percolation on a random regular graph [6, 7]. As mentioned, for our model $\phi_{\text{perc}} > 1/2 = p_{\text{perc}}$, due to the fact that persistent species are anticorrelated, tending not to be adjacent to one another.

E Subgraph emergence rule

To prove the result quoted in Sec 2.3 of the main text, we first prove a Lemma, which is interesting in its own right. We use the term "generically" for "with probability approaching one for large numbers of species".

Lemma (3): Consider a system with symmetric ($\alpha_{ij} = \alpha_{ji}$) and competitive ($\alpha_{ij} \geq 0$) interactions, sampled from some continuous distribution (such as a Gaussian distribution as in the main text). Suppose that the α_{ij} are changed continuously by shifting $m \equiv \text{mean}\,(\alpha_{ij})$ (other continuous shifts are also possible). Assume that the graph is feasible and stable in some range below $m = \alpha_c$, and not in a range above it. Then generically, it is feasibility that breaks at α_c, by a single species' abundance going to zero, while stability continues to hold.

Proof: We prove this by contradiction. Assume to the contrary that at $m = \alpha_c$ the graph becomes unstable. As the matrix α is symmetric it can be diagonalized. Let $\alpha = \sum_i \lambda_i \vec{v}_i \vec{v}_i^T$ be its eigen-decomposition, where T denotes the transpose operation, $\{\vec{v}_i\}$ are column eigenvectors, and λ_i the corresponding eigenvalues, with $\lambda_1 < \lambda_2 < ...$ (generically there is no degeneracy). Note that the values of quantities in this decomposition depend on m. These values are equilibrium solutions to Eq. 1 in the main text; from feasibility up to α_c, all $N_i > 0$ so,

$$\vec{N} = \alpha^{-1}\vec{u} = \Sigma_j \lambda_i^{-1} \vec{v}_i \vec{v}_i^T \vec{u} \ ,$$

where $\vec{u} = (1, 1, ..)$. By assumption, the system becomes unstable at α_c, $\lambda_1 \overset{m \to \alpha_c}{\longrightarrow} 0$. Since generically $\vec{v}_i^T \vec{u} \neq 0$, the first term dominates near α_c,

$$\vec{N} = \lambda_1^{-1} \vec{v}_1 \left(\vec{v}_1^T \vec{u}\right) + \langle \text{terms finite as } \alpha \to \alpha_c \rangle \ ,$$

so $\vec{N}$ diverges at $m \to \alpha_c$. Using $\alpha_{ij} \geq 0$ and feasibility, $N_i = 1 - \sum_j \alpha_{ij} N_j \leq 1$. Therefore, the divergence of the values of $\vec{N}$ must be towards $-\infty$, and so the N_i-values must cross zero at m *smaller than* α_c, in contradiction to the assumption. QED

Applying this lemma, a subgraph that loses feasibility at α_c generically does so by only one species having $N_i \to 0$. The remaining graph is still feasible and stable at α_c and for at least some range $[\alpha_c, \alpha_c + \varepsilon]$ above it

(because the stability and abundances of the remaining species change continuously). In the case of trees, removing a vertex splits the tree into multiple trees, see Fig 5 in the main text.

Without heterogeneity (when all $\alpha_{ij} = \alpha$) all trees have $\alpha_c \leq 1/2$, so it is interesting to consider the case where all the α_{ij} connecting to the extinct species N_i satisfy $\alpha_{ij} < 1/2$. In this case, the extinct species has $0 = N_i = 1 - \sum_j \alpha_{ij} N_j > 1 - C/2$, where C is the degree of species i, so $C > 2$ and the tree will split into at least three parts.

F Details of numerics

The python script used in order to run our simulations can be found in "https://github.com/stavmarcus/Local-and-collective-transitions-in-sparsely-interacting-ecological-communities". Each simulation used several randomly generated interaction matrices, and for each of them solved Eq. 1 in the main text for several random initial conditions, where the initial abundances were uniformly drawn from $[0, 1]$ .We use equal growth rates $r_i = 1$ and immigration rate $\lambda = 10^{-10}$, chosen to be small enough so that it does not affect the set of equilibria, but large enough to ensure invasions of extinct species with a positive growth rate within the run time. We have used an RK45 solver with relative and absolute tolerances of 10^{-12}, and used a run time of $5 \cdot 10^4$, which is much longer than the average time necessary to reach an equilibrium for the parameters that we use.

References

1. Horn RA, Johnson CR. Matrix Analysis. Cambridge University Press; 2013.
2. MacArthur R. Species Packing and Competitive Equilibrium for Many Species. Theoretical Population Biology. 1970 May;1(1):1-11.
3. May RM. Stability and Complexity in Model Ecosystems. Monogr Popul Biol. 1973;6:1-235.
4. da Fonseca CM, Petronilho J. Explicit Inverses of Some Tridiagonal Matrices. Linear Algebra and its Applications. 2001 Mar;325(1-3):7-21.
5. Bunde A, Havlin S, editors. Fractals and Disordered Systems. Berlin, Heidelberg: Springer Berlin Heidelberg; 1996.
6. Erdős P, Rényi A. On the Evolution of Random Graphs. Publ Math Inst Hung Acad Sci. 1960;5(1):17-60.
7. Joos F, Perarnau G. Critical Percolation on Random Regular Graphs. Proc Amer Math Soc. 2018 Mar;146(8):3321-32.
